# Social media enhances languages differentiation: a mathematical description

**DOI:** 10.1098/rsos.170094

**Published:** 2017-05-17

**Authors:** Ignacio Vidal-Franco, Jacobo Guiu-Souto, Alberto P. Muñuzuri

**Affiliations:** 1 Group of NonLinear Physics, Universidade Santiago de Compostela, 15706 Santiago de Compostela, Spain; 2 Department of Medical Physics, Universitary Hospital of Santiago de Compostela, 15706 Santiago de Compostela, Spain

**Keywords:** language competition, non-localized Turing structures, networks, differentiation induced by globalization

## Abstract

Understanding and predicting the evolution of competing languages is a topic of high interest in a world with more than 6000 languages competing in a highly connected environment. We consider a reasonable mathematical model describing a situation of competition between two languages and analyse the effect of the speakers' connectivity (i.e. social networks). Surprisingly, instead of homogenizing the system, a high degree of connectivity helps to introduce differentiation for the appropriate parameters.

## 1. Introduction

Thousands of languages have been competing worldwide for domination throughout history [1,2]. As a result of this competition/evolution, many of them have disappeared or been assimilated by more dominant languages. A homogenizing factor was introduced, mainly during the nineteenth century in Europe, when education became more popular and even mandatory. Its immediate effect was extinction or assimilation of minority languages. Nowadays, some of the less ‘popular’ languages face another problem, globalization of communications. Some authors assume that this new threat may increase suppression of minority languages [3].

Many attempts to mathematically model this complicated problem have been proposed in the literature [4–10]. Classical models predict extinction of either of two languages [11]. More recent models (Pinasco and Romanelli; Mira-Seoane) also include the possibility of exhibiting coexistence of the two languages. This is closer to real life, where languages manage to survive under very complicated situations and coexist with other more prominent languages.

More complicated models introduced the spatial distribution of speakers and tried to identify the conditions under which non-homogeneous distribution of languages may appear. The mechanism introduced by A. Turing in 1952 [12] to understand cell differentiation and morphogenesis can be considered in this context. Here, coexistence of two languages might be interpreted as the differentiation of a population of speakers. However, this implies cubic nonlinear models that most of the time do not correspond with physical mechanisms.

In this paper, we focus on the effect of social media on languages' competition and we address this effect with a completely different approach. We focus on the degree of connectivity between the different speakers and try to introduce it in our model.

We consider a non-diffusive non-local network of couplings (scale-free [13]) and analyse the effect of languages coexistence. This type of coupling, unlike a purely diffusive one, better reflects the degree of globalization of communications nowadays and the fact that speakers are connected not necessarily with other speakers located close to them but also with distant speakers via social media [14].

Details of the mathematical model used and the type of coupling are discussed in the next section. Then the numerical results are presented followed by a discussion and conclusions.

## 2. Numerical model

We consider a simple model describing the interaction of two competing languages, in particular, the predator–prey-like model proposed by Kandler & Steele [15] and Pinasco & Romanelli [16] whose equations are given by

2.1 $$\begin{array}{rl} \frac{\partial u}{\partial t} & =f(u,v)=u(a_{1}-b_{1}u-c_{1}v)\\ \mathrm{and}\;\frac{\partial v}{\partial t} & =g(u,v)=v(a_{2}-b_{2}v+c_{2}u), \end{array}\}$$

where *u* and *v* describe the number of speakers of each of the two competing languages considered. These equations describe the temporal evolution of both populations. The first two terms in equation (2.1) describe a logistic growth of independent populations (the coefficients *a*_1_ and *a*_2_ express the intrinsic growth rate of the number of speakers of each language, and *b*_1_ and *b*_2_ the self-limiting coefficients). The last term describes the influence between both languages with the conversion rates given by *c*_1_ and *c*_2_. In terms of the model [15,16], *c*_2_ can be interpreted as the status advantage of language 2. On the other hand, *c*_1_ is related to the effect that the status of language 2 has on the population 1. Both reflect the social and economic opportunities afforded to their speakers. In the absence of competition (i.e. *c*_1_ = *c*_2_ = 0), the model is reduced to a logistic growth of two independent populations. The results included in this paper considered *c*_1_ = *c*_2_.

The simplest way to introduce spatial distribution of the two populations is considering a diffusion term in equation (2.1) [15,16] such as

2.2 $$\begin{array}{rl} \frac{\partial u}{\partial t} & =u(a_{1}-b_{1}u-c_{1}v)+D_{u}\nabla^{2}u\\ \mathrm{and}\;\frac{\partial v}{\partial t} & =v(a_{2}-b_{2}v+c_{2}u)\;+D_{v}\nabla^{2}v, \end{array}\}$$

where *D_u_* and *D_v_* are the diffusion coefficients for both species that describes the normal movement of a given population from higher-density regions to lower-density regions [17]. Note that now *u* and *v* describe the number of speakers of each language at each location. Diffusive coupling indicates that speakers just interact with close neighbours as was the case before social media or long-distance communications became popular. This model has been analysed and it was shown that it is not enough to show Turing structures and thus describe a non-homogeneous stationary solution.

Recently, cross-diffusion has been considered in this context and proved enough to obtain Turing structures. Cross-diffusion for the minority population (*u* in our case) expresses the flux of such a population because of the presence of the dominant population (and vice versa). The mathematical form of such a model is [18,19]

2.3 $$\begin{array}{rl} \frac{\partial u}{\partial t} & =u(a_{1}-b_{1}u-c_{1}v)+\nabla^{2}[(d_{1}+a_{11}u+a_{12}v)u]\\ \mathrm{and}\;\frac{\partial v}{\partial t} & =v(a_{2}-b_{2}v+c_{2}u)+\nabla^{2}[(d_{2}+a_{21}u+a_{22}v)v], \end{array}\}$$

where *d*_1_ (respectively, *d*_2_) corresponds with the normal diffusion coefficient for the *u*-variable (*v* respectively). The positive constants *a*_12_ and *a*_21_ are referred to as cross-diffusion coefficients, which describe that the minority language tends to avoid higher density of the dominant and vice versa by diffusing away. This feature clearly applies to language competition as has been previously considered in different competition contexts [18,19]. In addition, for the dominant and the minority speakers, the positive constants *a*_11_ and *a*_22_ are self-diffusion rates due to pressure within their own species. For the results presented in this paper, we always considered *a*_11_ = *a*_22_ = 0.

Equation (2.3) with the appropriate parameter values are shown to exhibit Turing structures, i.e. stationary non-homogeneous solutions [18,19] that within this context can be understood as the coexistence of two competing languages.

Another important feature in language competition in the present times is the connectivity of the speakers. In former times, the type of connectivity between speakers could be appropriately modelled by diffusion as in equation (2.3) (speakers are only connected and, thus, influenced by neighbouring speakers). Now, speakers are highly connected with others worldwide through social media. This high degree of connectivity cannot be described by diffusion anymore and complex networks should be considered to model them [20–23].

In order to introduce a network in our system, we need to consider that the speakers instead of being geographically distributed with a Cartesian metrics occupy discrete nodes of a network and are transported over links connecting them. We consider a network of *N* nodes and each node has a degree of connectivity (number of connections) given by *k_i_*. Thus, the minority-language-speakers' amount in node *i* is given by *u_i_* (respectively, *v_i_* for the dominant language). Equations describing network-organized competition between languages with cross-diffusion are thus given by

2.4 $$\begin{array}{rl} \frac{du_{i}}{dt} & =u_{i}(a_{1}-b_{1}u_{i}-c_{1}v_{i})+\overset{N}{\underset{i=1}{\sum }}L_{ij}[(d_{1}+a_{11}u_{j}+a_{12}v_{j})u_{j}]\\ \mathrm{and}\frac{dv_{i}}{dt} & =v_{i}(a_{2}-b_{2}v_{i}+c_{2}u_{i})+\overset{N}{\underset{i=1}{\sum }}L_{ij}[(d_{2}+a_{21}u_{j}+a_{22}v_{j})v_{j}], \end{array}\}$$

with *i* = 1 … *N*. *L_ij_* is the network Laplacian matrix given by

2.5 $$L_{ij}=A_{ij}-k_{i}\delta_{ij}.$$

The topology of the network is defined by a symmetric adjacent matrix *A_ij_* defined as

2.6 $$A_{ij}=\left\{ \begin{array}{ll} 1 & \text{if node }\;i\;\text{ and }\;j\;\text{ are connected }(i,j=1\ldots N)\\ 0 & \mathrm{otherwise}. \end{array}\right.$$

In this paper we consider a 1000 nodes scale-free network [12]. Scale-free networks are characterized by a power-law connectivity degree distribution [12,24,25] and have been demonstrated to be the type of connectivity distribution associated with the World Wide Web, social media and neuronal networks among others [14]; in the following, we assume that a population of speakers fits well this type of network. In general, the probability of having a node with connectivity *k* is given by *k*^−*γ*^ (in our case *γ* = 3, following Turing [12]). We use the mean value of *k*, ⟨k⟩, across the entire network to assess the overall connectivity. It is important to notice that, while ⟨k⟩ can be modified at the network generation step, allowing us to study networks with different connectivity, the value of *γ* always remains the same, as this is a property inherent to scale-free networks built following Turing [12].

Systems with a network type of connectivity are also known to exhibit stationary non-homogeneous patterns, Turing patterns in network-organized systems [26,27]. Now, Turing patterns are not characterized by a characteristic wavelength, because there is no spatial metrics in a scale-free network. Nevertheless, there are still two different states and differentiation happens; some of the nodes will evolve to a completely different state than the others. McCullen & Wagenknecht [27] and Wolfrum [28] provide detailed analysis of the different stationary patterns such systems may exhibit.

Equation (2.4) with the scale-free network defined by equations (2.5) and (2.6) can be analysed by linear stability analysis following Nakao & Mikhailov [26]. The results show that these non-localized Turing patterns may exist in our model (see appendix A for details) for the appropriate parameter values.

Numerical simulations of the above equations were performed using a fourth-order Runge–Kutta algorithm. The model parameters used are: *a*_1_ = 1, *a*_2_ = 2, *b*_1_ = 0.08, *b*_2_ = 0.15, *c*_1_ = *c*_2_ = 0.05, and the diffusion coefficients were set as follows: *d*_1_ = 0.001, *d*_2_ = 0.001 (small normal diffusion), *a*_11_ = *a*_22_ = 0 (no self-diffusion rate) and *a*_12_ = 0; *a*_21_ was the cross-diffusion control parameter and varied for the different simulations presented in this paper.

The scale-free network of couplings connecting the nodes was obtained following the Albert–Barabasi algorithm [12,14]. Different networks with different average connectivity ⟨k⟩ where considered in the paper.

## 3. Results

**Figure 1.**
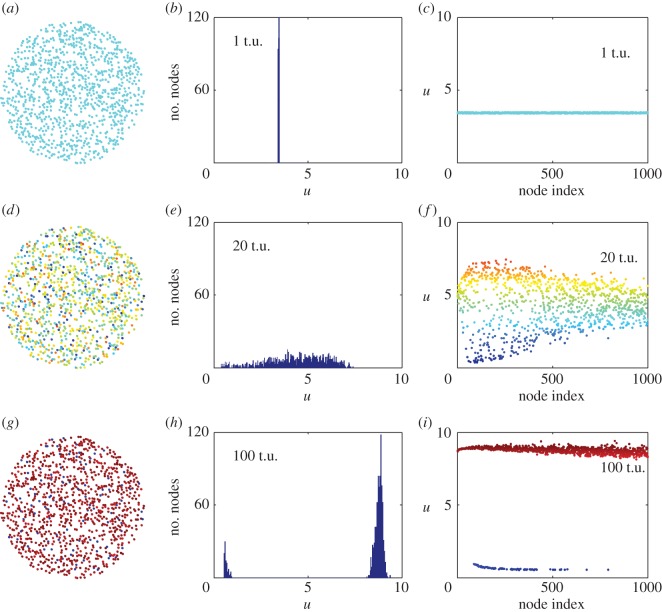
Temporal evolution of equation (2.4) in a scale-free network (*N* = 1000 nodes with an average degree of coupling ⟨k⟩=50). First column is a graph with the nodes and colours corresponding with the value of *u*-variable for each node. Second column plots the histogram distribution of the values of the *u*-variable for each node. Third column plots the value of the *u*-variable for each node. The first row (panels *a*, *b* and *c*) shows the simulations at *t* = 1 t.u. (arbitrary temporal units), second row (panels *d*, *e* and *f*) at *t* = 20 t.u. and third row (panels *g*, *h* and *i*) at *t* = 100 t.u. when a stationary solution is achieved. Model parameters: *a*_1_ = 1, *a*_2_ = 2, *b*_1_ = 0.08, *b*_2_ = 0.15, *c*_1_ = *c*_2_ = 0.05; diffusion related coefficients: *d*_1_ = *d*_2_ = 0.001, *a*_11_ = *a*_22_ = 0, *a*_12_ = 0 and *a*_21_ = 0.06. Nodes are ordered according to their connectivity degrees.

**Figure 2.**
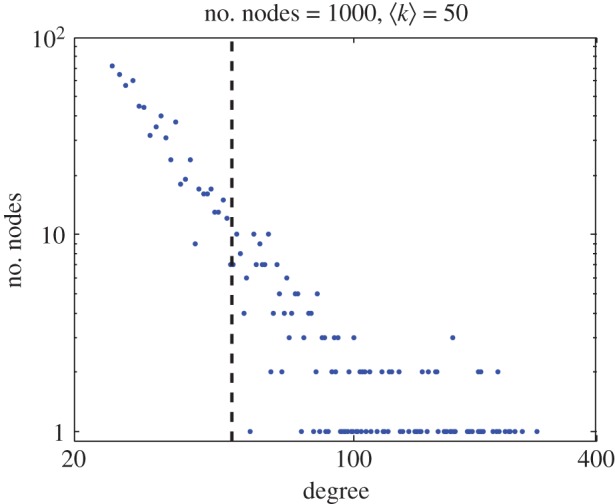
Degree distribution for computations shown in figure 1. *N* = 1000 nodes and the average connectivity ⟨k⟩=50 in logarithmic scale. Note that it follows a power law characteristic of scale-free networks with an exponent −3.

Numerical simulations of equation (2.4) with initial conditions around the steady-state result in a non-localized Turing structure are plotted in figure 1. The first column is a graph with the nodes, colours corresponding with the value of the *u*-variable for each node. The second column plots the histogram distribution of the values of the *u*-variable for each node. The third column plots the value of the *u*-variable for each node. Nodes with higher connectivity occupy lower indices and vice versa. The first row corresponds with the state of the network at *t* = 1 t.u. (arbitrary temporal unit). Here, all nodes are still close to the steady state with some random fluctuations. As time evolves, also the values of the variables for each node start differentiating as seen in the second row for *t* = 20 t.u. The nodes with smaller index (corresponding to those with higher connectivity) start differentiating before those with large index and low connectivity. The last row presents the stationary configuration at *t* = 100 t.u. Nodes split into two groups with completely different values for the variables. This state is a non-localized Turing structure and once formed remains stable as long as no new perturbation is introduced in the system. The average connectivity in the network used for the simulation in figure 1 is ⟨k⟩=50. Figure 2 shows the connectivity degree distribution typical of a scale-free network. The results plotted in this figure demonstrate that a population initially close to the stationary state spontaneously evolves into a set of two clearly differentiated populations once a network is considered. Those individuals more connected are more likely to differentiate than those with low connectivity.

**Figure 3.**
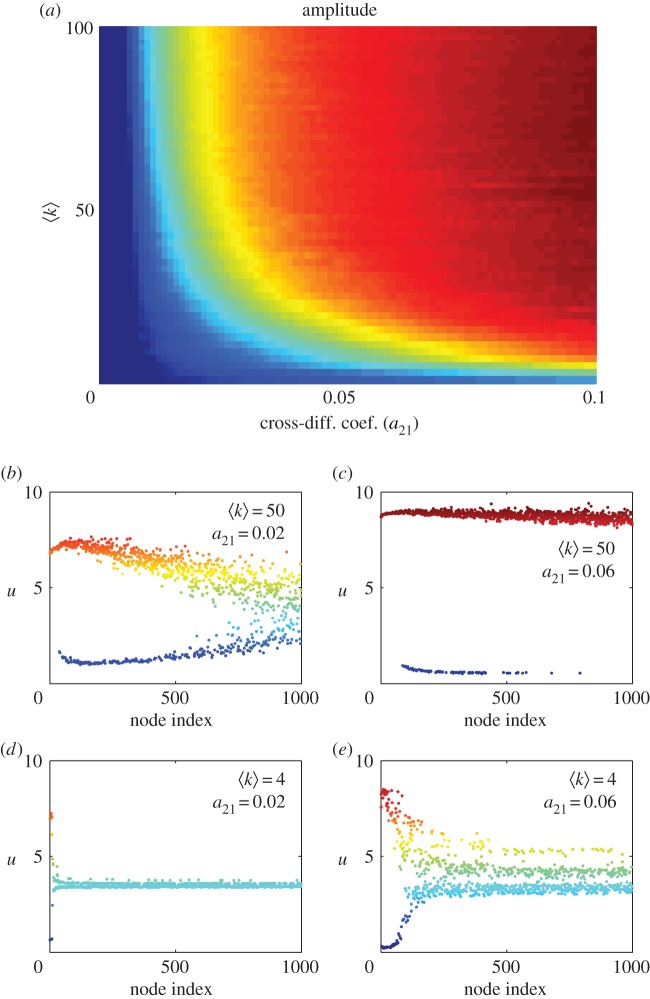
(*a*) ⟨k⟩-*a*_21_ phase diagram. Amplitude calculated as the difference between the largest value of *u*-variable minus the smallest. Small values of the amplitude are plotted in blue while larger values are in red. Areas in dark red correspond to values of the parameters where nodes differentiate via Turing mechanism. Different values of *u*-variables for all nodes for different cases; (*b*) ⟨k⟩=50 and *a*_21_ = 0.02, differentiation for small values for the connectivity although nodes with small connectivity (high node index) still fail to differentiate. (*c*) ⟨k⟩=50 and *a*_21_ = 0.06, differentiation via Turing mechanism. (*d*) ⟨k⟩=4 and *a*_21_ = 0.02, no differentiation. (*e*) ⟨k⟩=4 and *a*_21_ = 0.06, small differentiation in those nodes with high connectivity. Nodes are ordered according to their connectivity degrees.

The influence of the cross-diffusion coefficient, *a*_21_, and the average degree of connectivity, ⟨k⟩, on the system is analysed in figure 3. The coefficient *a*_21_ accounts for the degree of repulsion of the minority population by the dominant one, while ⟨k⟩ is directly connected to the importance of the social media in the population. A direct measurement of said differentiation is given by the Turing structure amplitude or the difference between the largest value of the *u*-variable minus the smallest. Figure 3*a* is a phase diagram where the amplitude of the structure is plotted for each value of our control parameters, *a*_12_ and ⟨k⟩. Red areas correspond to the largest amplitude of the Turing pattern (largest differentiation) while regions in dark blue correspond to zero amplitude (i.e. no differentiation). Some minimum value for *a*_21_ is needed to achieve differentiation but once this threshold is crossed the larger the average connectivity of the network ⟨k⟩ the larger differentiation is achieved. Note that, although differentiation was also observed in purely diffusive networks with cross-diffusion [18,19], considering a complex network coupling drastically lowered the required magnitude of the diffusion coefficients for the differentiation to occur. In this sense, we can affirm that social media, represented by complex network type of coupling, increases the possibilities to obtain spatial coexistence of both languages.

The following panels in figure 3 present the stationary configuration (at *t* = 2500 t.u.) of the nodes for different values of the parameters in the phase diagram. Note that for small values of ⟨k⟩ and *a*_21_ (figure 3*d*) no differentiation takes place, all nodes remain at a value close to the stationary state (meaning that no coexistence of languages is expected). For this small value of the average connectivity (⟨k⟩=4) and increasing *a*_21_ (figure 3*e*), some small differentiation is observed but only on those nodes with larger degree of connectivity (small node index). This effect is more clearly seen keeping constant *a*_21_ = 0.02 and increasing ⟨k⟩ till 50. This case is shown in figure 3*b*; now two independent states are clearly differentiated although, again, nodes with small connectivity (high node index) fail to differentiate. Figure 3*c* shows a state where all nodes divide into two well-differentiated states.

## 4. Discussion and conclusion

Based on a simple predator–prey mechanism, we consider the effect of non-local coupling between speakers. This effect was included into the equations considering a scale-free network that has been proved to be suitable to represent this type of social network. This model is used to analyse the effect of connectivity between speakers (i.e. via social media) in language differentiation.

The results obtained are clear: speakers highly connected are more likely to differentiate and move to a different state. The mechanism that triggers differentiation is based on Turing instability. Classical Turing instability is induced by diffusion in diffusive networks; the whole system is in a steady state (stable without diffusion) but small perturbations trigger the system out of the steady state and force the system into a new situation where two completely different values coexist. In our case (scale-free networks), the instability is triggered by the degree of connectivity between the nodes. Thus, those nodes more connected are more likely to accumulate perturbations from their connections and move to one of the two stable values.

In terms of a bilinguals model, this means that speakers with high connectivity (high activity in social media) are more likely to differentiate or keep differentiated. Once the final state is achieved, the situation remains unchanged, thus predicts coexistence of both languages. Our results show that this is only possible if the overall connectivity exceeds a certain threshold.

One can, thus, conclude that social media (or any kind of activity that increases connectivity between speakers) in a scale-free network helps to prevent homogenization when two competing languages are considered.

## Figures and Tables

**Figure 4.**
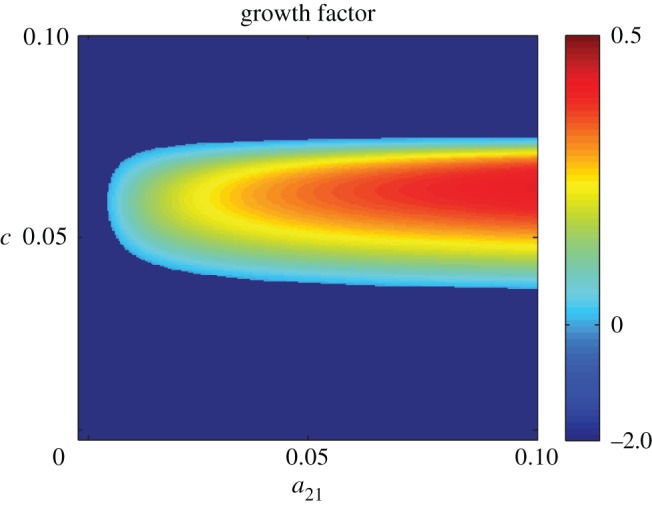
Growth factor calculated for different values of the cross-diffusion coefficient (*a*_21_) and the status coefficient *c* (*c* = *c*_1_ = *c*_2_). Areas in dark blue correspond to negative values, thus no Turing structures appear. Turing structures only appear for growth factors larger than 0 as those plotted in colours. (Model parameters: *a*_1_ = 1, *a*_2_ = 2, *b*_1_ = 0.08, *b*_2_ = 0.15, *d*_1_ = 0.001, *d*_2_ = 0.001, *a*_11_ = *a*_22_ = 0).

## Data accessibility

Numerical data presented in this paper were obtained by direct numerical simulation of the equations presented here. Any commercial or free software for PDE solving might render the same data. In any case, our code and data are available from the Dryad Digital Repository: http://dx.doi.org/10.5061/dryad.b2207 [29].

## Appendix A. Linear stability analysis

We consider the set of equations given by (2.4) with the Laplacian matrix defined as in equations (2.5) and (2.6) describing the cross-diffusion terms in a scale-free network. We performed a linear stability analysis following Nakao & Mikhailov [26] in order to determine the growth rate of each mode but still considering the cross-diffusion terms. The linearized version of equation (2.4) is

A 1 $$\left(\begin{array}{c} \frac{du_{i}}{dt}\\ \frac{dv_{i}}{dt} \end{array}\right)=J{|}_{u_{0},v_{0}}\left(\begin{array}{c} u_{i}\\ v_{i} \end{array}\right)+\overset{N}{\underset{i=1}{\sum }}L_{ij}\left(\begin{array}{cc} d_{1}+a_{11}u_{0}+a_{12}v_{0} & a_{12}u_{0}\\ a_{21}v_{0} & d_{2}+2u_{0}+a_{22}v_{0} \end{array}\right)\left(\begin{array}{c} u_{j}\\ v_{j} \end{array}\right)$$

or, expressed in a more compact notation:

A 2 $$u_{t}^{i}=J{|}_{u_{0},v_{0}}\cdot u^{i}+\overset{N}{\underset{i=1}{\sum }}L_{ij}D{|}_{u_{0},v_{0}}\cdot u^{j},$$

where **u **= (*u*, *v*) is a vector whose two components are the two variables of the model, (*u*_0_, *v*_0_) is the steady state (stable without influence of the network), $J{|}_{u_{0},v_{0}}$ is the Jacobian matrix and $D{|}_{u_{0},v_{0}}$ is the linearized diffusion matrix, both evaluated at the fixed point (*u*_0_, *v*_0_) The evolution of a perturbation to the fixed point can be evaluated through the eigenvalues ($\Lambda_{\alpha }$) of the Laplacian matrix, defined in our case by,

A 3 $$\overset{N}{\underset{i=1}{\sum }}L_{ij}\phi_{i}^{\left(\alpha \right)}=\Lambda_{\alpha }\phi_{i}^{\left(\alpha \right)}.$$

The perturbations to each node can be expressed as $\delta u_{i}=\sum_{\alpha =1}^{N}C_{\alpha }e^{\lambda_{\alpha }t}\phi_{i}^{\left(\alpha \right)}$ and $\delta v_{i}=\sum_{\alpha =1}^{N}C_{\alpha }B_{\alpha }e^{\lambda_{\alpha }t}\phi_{i}^{\left(\alpha \right)}$. Substituting in equation (A 1) we obtain

A 4 $$\lambda_{\alpha }\left(\begin{array}{c} 1\\ B_{\alpha } \end{array}\right)=\left(\begin{array}{cc} f_{u}+D_{11}\Lambda_{\alpha } & f_{v}+D_{12}\Lambda_{\alpha }\\ g_{u}+D_{21}\Lambda_{\alpha } & g_{v}+D_{22}\Lambda_{\alpha } \end{array}\right)\left(\begin{array}{c} 1\\ B_{\alpha } \end{array}\right)=M\left(\begin{array}{c} 1\\ B_{\alpha } \end{array}\right)$$

The growth rates $\lambda_{\alpha }$ for each mode can be determined by $\mathrm{Det}(M-I\lambda_{\alpha })=0$, which leads to the following characteristic equation:

A 5 $$\lambda_{\alpha }^{2}-(\mathrm{Tr}(J)+\mathrm{Tr}(D)\Lambda_{\alpha })\lambda_{\alpha }+\mathrm{Det}(J)+\mathrm{Det}(D)J\cdot D^{-1}\Lambda_{\alpha }+\mathrm{Det}(D)\Lambda_{\alpha }^{2}=0.$$

The condition for Turing instability is given by the existence of at least one eigenvalue ($\Lambda_{\alpha }$) with a growth rate ($\lambda_{\alpha }$) larger than zero. An illustration of this is in figure 4. Here, the maximum growth factor is plotted for different values of the cross-diffusion coefficient (*a*_21_) and the status coefficient *c* (*c* = *c*_1_ = *c*_2_) showing the region in the parameter space where non-localized structures may appear.
